# Intestinal Parasitic Infections and Respiratory Morbidity in Children with Sickle Cell Disease in French Guiana: A Multicenter Cross-Sectional Study

**DOI:** 10.3390/tropicalmed11070195

**Published:** 2026-07-13

**Authors:** Gabriel Bafunyembaka, Christian Kinsiona, Joody Mafema, Emmanuel Irakoze, Philbert Furero, Christelle Samou, Narcisse Elenga

**Affiliations:** 1 Department of Pediatrics, Centre Hospitalier de l’Ouest Guyanais (CHOG), “Franck Joly” Hospital, 1465 Boulevard de la Liberté, Saint-Laurent-du-Maroni 97320, French Guiana; p.furero@chu-guyane.fr (P.F.); c.samou-fantcho@chu-guyane.fr (C.S.); 2 Doctoral School, University of French Guiana, Campus de Troubiran, 2091 Route de Baduel, Cayenne 97300, French Guiana; elengafr@yahoo.fr; 3 Department of Pediatrics, Centre Hospitalier de Kourou, CHU de Guyane—Site de Kourou, Avenue Léopold Héder, Kourou 97387, French Guiana; kinsionachristian2014@gmail.com; 4 Department of Pediatrics, Centre Hospitalier Andrée-Rosemon, 3 Avenue Alexis Blaise, Cayenne 97300, French Guiana; joody.mafemamissindu@ch-cayenne.fr (J.M.); emmanuel.irakoze@ch-cayenne.fr (E.I.)

**Keywords:** sickle cell disease, intestinal parasites, helminths, asthma, children, French Guiana, bronchial obstruction, acute chest syndrome

## Abstract

Background: Intestinal parasitic infections are common in tropical settings and may influence immune regulation, allergic responses, and respiratory health. In children with sickle cell disease (SCD), respiratory morbidity is multifactorial, and the contribution of intestinal parasites remains uncertain. This study assessed whether documented intestinal parasitic infections, particularly helminth infections, were associated with asthma-like respiratory phenotypes and clinical morbidity in children with SCD in French Guiana. Methods: We conducted a multicenter cross-sectional analysis of children and adolescents younger than 18 years with confirmed SCD who were being followed by the pediatric SCD centers of Saint-Laurent-du-Maroni, Cayenne, and Kourou between January 2022 and December 2025. Clinical, respiratory, and parasitological data were extracted from routine-care medical records. Intestinal parasitic infection was defined as at least one documented stool parasitology result identifying *Strongyloides stercoralis*, hookworm, or *Entamoeba histolytica*, according to the variables consistently available in the database. Respiratory outcomes included physician-diagnosed asthma, bronchial obstruction, bronchodilator reversibility, and an asthma-like respiratory phenotype. Results: Among 233 included children, 105 (45.1%) had at least one documented intestinal parasitic infection and 82 (35.2%) had a helminth infection. Hookworm was the most common parasite (75/233, 32.2%), followed by *Entamoeba histolytica* (33/233, 14.2%) and *Strongyloides stercoralis* (24/233, 10.3%). An asthma-like respiratory phenotype was observed in 32/105 infected children (30.5%) versus 44/128 non-infected children (34.4%). Bronchial obstruction and bronchodilator reversibility were also similar between groups. After adjustment for age, sex, genotype, hydroxyurea treatment, rural residence, site of follow-up, and environmental tobacco smoke exposure, intestinal parasitic infection was not associated with asthma-like respiratory phenotype (adjusted OR: 0.94, 95% CI: 0.51–1.72; *p* = 0.844), recurrent hospitalization, or history of acute chest syndrome. Conclusions: In this routine-care multicenter pediatric SCD cohort from French Guiana, intestinal parasitic infections were common but were not associated with respiratory phenotype or selected morbidity outcomes. Because testing and respiratory assessment were not systematic, these findings should be interpreted as showing that routine parasitological status alone could not identify children with respiratory morbidity. Prospective studies with standardized repeated parasitological, immunological, environmental, and respiratory assessments are needed.

## 1. Introduction

Sickle cell disease (SCD) is a major inherited hemoglobin disorder and remains an important cause of pediatric morbidity worldwide, particularly in populations of African, Caribbean, Mediterranean, Middle Eastern, and Indian ancestry [1]. In France, SCD represents a substantial public health burden, with an important concentration of affected individuals in overseas territories, including French Guiana [2]. In French Guiana, SCD has the highest reported incidence in France, with approximately one affected newborn per 200 births, and it remains a major public health issue in pediatric practice.

Respiratory morbidity is a central component of the clinical burden of SCD in children. It is more common in this population because hemolysis, chronic inflammation, impaired nitric oxide bioavailability, recurrent vaso-occlusion, infection, hypoxemia, and acute chest syndrome (ACS) can affect the airways and pulmonary vasculature. Children with SCD may therefore experience recurrent wheezing, cough, dyspnea, lower airway obstruction, bronchodilator reversibility, ACS, and progressive pulmonary complications [3,4]. In routine clinical practice, asthma and SCD-related respiratory disease may be difficult to distinguish because respiratory symptoms can overlap with ACS, infection, vaso-occlusive pain, and sickle cell-related lung injury [5].

For this reason, we used the pragmatic term “asthma-like respiratory phenotype” to describe children with physician-diagnosed asthma and/or objective airway involvement recorded during routine care. This term was not intended to replace a standardized asthma diagnosis or to include ACS itself as a diagnostic criterion; rather, it captured overlapping respiratory manifestations in a retrospective clinical dataset.

In tropical environments, intestinal parasitic infections remain common and may represent an important but underexplored environmental determinant of immune and respiratory health. Soil-transmitted helminths and other intestinal parasites are strongly linked to poverty, inadequate sanitation, humid climates, and rural or peri-urban living conditions [6,7]. In children, these infections may contribute to anemia, nutritional impairment, chronic inflammation, immune modulation, and altered gut microbial ecology. These mechanisms may be particularly relevant in SCD, where baseline inflammation, hemolysis, endothelial dysfunction, and susceptibility to hypoxemia already contribute to clinical vulnerability.

The relationship between intestinal parasites and asthma or allergic disease is complex and remains debated. Helminth infections can modulate immune responses through Th2 polarization, eosinophilia, IgE production, regulatory T-cell activation, and anti-inflammatory cytokines such as IL-10 and TGF-beta. Depending on parasite species, parasite burden, timing of infection, chronicity, and host background, helminths may either promote allergic inflammation or downregulate atopic responses [8].

Some specific parasites may have particular respiratory relevance. Strongyloides stercoralis is a soil-transmitted helminth capable of chronic infection through autoinfection and is endemic in many tropical and subtropical regions [9,10]. Hookworm infections may contribute to anemia and nutritional deficiency, potentially worsening the clinical burden of children with SCD. Protozoan infections such as Entamoeba histolytica may also indicate fecal-oral environmental exposure and poor sanitation, even if their direct relationship with asthma-like respiratory phenotypes is less well established.

Despite these plausible links, very few studies have specifically investigated intestinal parasitic infections in relation to asthma-like respiratory phenotypes among children with SCD. The most relevant research gap concerns soil-transmitted helminths, particularly *Strongyloides stercoralis* and hookworm, because these parasites may influence eosinophilia, anemia, immune regulation, corticosteroid safety, and pulmonary symptoms. This question is particularly important in tropical territories such as French Guiana, where helminths, allergic sensitization, humidity, mold exposure, and social vulnerability may coexist.

The present study was therefore designed to evaluate the association between documented intestinal parasitic infections and asthma-like respiratory phenotypes in children and adolescents with SCD followed in French Guiana. We hypothesized that intestinal parasitic infections, particularly helminth infections such as *Strongyloides stercoralis* and hookworm infections, may be associated with respiratory phenotypes or clinical morbidity in this vulnerable population. Secondary objectives were to explore whether parasite status was associated with confirmed asthma, bronchial obstruction, bronchodilator reversibility, recurrent hospitalization, or history of ACS.

## 2. Methods

### 2.1. Study Design and Setting

We conducted a multicenter cross-sectional study among children and adolescents with SCD followed in French Guiana between January 2022 and December 2025. Data were collected from the three participating pediatric SCD centers: Centre Hospitalier de l’Ouest Guyanais in Saint-Laurent-du-Maroni, Centre Hospitalier Andrée-Rosemon in Cayenne, and Centre Hospitalier de Kourou. Clinical, respiratory, and parasitological information was extracted from routine-care medical records during this study period.

### 2.2. Study Population

Children and adolescents were defined as patients younger than 18 years at the time of clinical assessment. Patients with confirmed SCD were eligible if they had available clinical, respiratory, and parasitological data during the study period. SCD diagnosis was based on hemoglobin electrophoresis, high-performance liquid chromatography, or a documented diagnosis in the medical record. Children with missing parasitological data were excluded from the main analysis.

During the same study period, the broader multicenter pediatric SCD follow-up population in French Guiana comprised approximately 588 children. The present analytic sample was restricted to the 233 children for whom both parasitological data and respiratory/asthma-related evaluation were available within this period. Therefore, this study should be interpreted as an analysis of a clinically assessed subset rather than a systematic screening of the entire pediatric SCD population.

Group assignment was based on documented routine-care parasitological results. Stool parasite testing was not performed as a systematic population-wide screening program for all children with SCD. It could be requested in symptomatic children, particularly in the presence of gastrointestinal symptoms, anemia, eosinophilia, nutritional concerns, or suspected environmental exposure, but could also be performed in asymptomatic children according to local clinical practice in this tropical Amazonian setting. Consequently, the comparison group consisted of children with no documented intestinal parasite on available routine-care stool parasitology, not children proven to be parasite-free by repeated systematic screening.

### 2.3. Main Exposure: Intestinal Parasitic Infection

The main exposure was the presence of at least one intestinal parasitic infection detected during routine parasitological assessment. The parasites considered in the analysis were limited to the organisms consistently recorded in the study database and clinically reviewed during data extraction: *Strongyloides stercoralis*, hookworm, and *Entamoeba histolytica*. Other protozoa or helminths were not included because they were not consistently available across centers in the structured dataset.

Parasitological assessment was performed in routine hospital laboratories using standard stool ova-and-parasite examination. According to local laboratory practice and clinical indication, this included direct microscopic examination of fresh or preserved stool samples and concentration techniques. For suspected *Strongyloides stercoralis* infection, larval detection methods such as Baermann-type concentration or other locally available techniques could be used when requested. Molecular assays were not systematically performed during the study period. The term “routine parasitological assessment” therefore refers to stool parasitology ordered as part of routine clinical care and interpreted by hospital laboratories, rather than a standardized research screening protocol.

Because parasitological testing was based on routine-care stool examinations, the number of stool samples, timing of testing, and use of complementary techniques were not standardized across children or centers. A single available stool examination may have limited sensitivity for light or intermittent helminth infections and may therefore have caused false-negative exposure misclassification. Quantitative egg counts, larval burden, molecular confirmation, and standardized repeated stool sampling were not available. Consequently, negative parasitological status should be interpreted as no documented parasite in available routine-care records rather than definite absence of infection.

### 2.4. Exposure Coding and Species-Specific Descriptive Approach

- Any intestinal parasitic infection, defined as the presence of at least one recorded intestinal parasite.
- Helminth infection, defined as the presence of *Strongyloides stercoralis* and/or hookworm.

Children with no documented intestinal parasitic infection in the available stool parasitology records were used as the reference group. Descriptive analyses reported the frequencies of hookworm, *Strongyloides stercoralis*, and *Entamoeba histolytica* separately. Robust species-specific multivariable models were not prespecified as primary analyses because the study was not powered for small parasite-specific subgroups and because coinfections and sparse outcome cells could lead to unstable estimates.

### 2.5. Outcomes

The primary outcome was an asthma-like respiratory phenotype, defined pragmatically using available clinical and functional data. This composite outcome included physician-diagnosed asthma recorded in the medical file and/or objective airway involvement, including bronchial obstruction or bronchodilator reversibility. Confirmed asthma was analyzed both as one component of this composite outcome and as a separate outcome, which explains why similar counts may be observed when most children meeting the composite definition also had a physician diagnosis of asthma.

Respiratory evaluation was also performed as part of routine care rather than as universal screening. Spirometry was generally performed in children old enough to provide reliable lung function measurements and/or when respiratory symptoms or clinical concerns were present, including recurrent wheezing, cough, dyspnea, suspected asthma, previous acute chest syndrome, or suspected airway obstruction. Physician-diagnosed asthma corresponded to asthma recorded in the medical file by the treating clinician, supported by clinical history and lung function testing when available.

- Confirmed asthma, defined as physician-diagnosed asthma recorded in the medical file;
- Bronchial obstruction, defined according to the available spirometry interpretation, with FEV1/FVC < 80% used as a pragmatic threshold when the lower limit of normal was unavailable;
- Bronchodilator reversibility, defined as an increase in FEV1 of at least 12% after bronchodilator administration when available.

Secondary clinical outcomes included recurrent hospitalization, defined as more than two hospitalizations during the available follow-up period, and history of acute chest syndrome. History of ACS was assessed from the medical record and retained when a previous clinician-documented episode was recorded, using usual pediatric SCD criteria combining an acute respiratory illness with a new pulmonary infiltrate and/or oxygen requirement, fever, chest pain, cough, wheezing, or respiratory distress.

### 2.6. Covariates

Potential confounders were selected based on clinical relevance and availability in the routine-care database. The main covariates considered were age, sex, SCD genotype, hydroxyurea treatment, rural or urban residence, site of follow-up, environmental tobacco smoke exposure, and allergen sensitization. Variables reflecting SCD severity or respiratory risk, such as baseline hemoglobin, prior ACS frequency, asthma treatment, corticosteroid exposure, eosinophil count, and detailed environmental exposures, were not consistently available and therefore could not be included in the primary models.

Rural residence was defined according to the child’s recorded place of residence and local catchment classification, distinguishing children living in rural or remote municipalities and villages from those living in the main urban coastal areas. Allergen sensitization was defined as at least one positive skin prick test or documented specific IgE result when available in the clinical record. These variables were used as contextual covariates rather than as exhaustive measures of environmental exposure.

Additional household-level variables requested by peer review, including sanitation, household crowding, nutritional status, detailed socioeconomic deprivation, and previous deworming history, were not systematically available in the retrospective database and could not be included in the multivariable models.

### 2.7. Data Collection

Data were extracted from a structured clinical database completed during routine pediatric follow-up and cross-checked with medical records when needed. The database included demographic characteristics, SCD genotype, hydroxyurea treatment, place of residence, respiratory history, spirometry results when performed, bronchodilator reversibility results when available, allergen sensitization data when available, hospitalization history, ACS history, and stool parasitology results. Data consistency was reviewed before analysis, and implausible or duplicate entries were checked against source records. All data were anonymized before analysis, and each participant was assigned a unique study identifier.

### 2.8. Statistical Analysis

Categorical variables were described as frequencies and percentages. Continuous variables were described using means and standard deviations or medians and interquartile ranges, depending on their distribution. Children with and without intestinal parasitic infection were compared using chi-square or Fisher exact tests for categorical variables and Student *t*-test or Mann–Whitney U test for continuous variables, as appropriate.

Logistic regression was used to estimate crude odds ratios (ORs) and 95% confidence intervals (CIs) for the association between intestinal parasitic infection and respiratory or clinical outcomes. Multivariable logistic regression was used to estimate adjusted odds ratios (aORs), controlling for age, sex, SCD genotype, hydroxyurea treatment, rural residence, site of follow-up, and environmental tobacco smoke exposure. Penalized logistic regression using Firth correction was considered if sparse-data bias or complete separation was detected. All statistical analyses were performed using Stata version 17.

Exploratory stratified descriptions were performed by age group and by SCD genotype group, when cell sizes allowed. Because of the limited subgroup sample sizes and sparse outcome events, separate age- or genotype-stratified regression models were considered statistically unstable and were not used for definitive inference.

No a priori sample-size calculation was performed because this was a retrospective analysis of all eligible children with available parasitological and respiratory data during the January 2022 to December 2025 study period. With 105 infected and 128 non-infected children, the study had limited power to detect very small between-group differences. Based on the observed asthma-like respiratory phenotype rates of 30.5% and 34.4%, negative findings should be interpreted cautiously and should not be considered definitive evidence of absence of association.

### 2.9. Missing Data

For each analysis, denominators were reported according to available data. Complete-case analysis was used for multivariable models. Variables with substantial missing data were excluded from the primary adjusted models or considered only in exploratory analyses.

### 2.10. Ethical Considerations

This study used anonymized data collected during routine clinical care. It was conducted within the DREPASTHME retrospective research framework (version 1.0, 22 March 2024), with the Centre Hospitalier de Cayenne as data controller, Professor Narcisse Elenga as scientific lead, and support from CIC Antilles-Guyane INSERM CIC 1424. According to French regulations, retrospective analyses of existing routine-care data that do not involve any interventions in participants may be conducted under the MR-004 reference methodology, provided that data protection, information, and opposition requirements are respected. The study therefore did not require individual written consent or a separate prospective ethics committee approval; the waiver of written consent was based on the retrospective anonymized routine-care design and applicable French regulatory provisions. No individual-level identifying information was included in the analytic dataset.

## 3. Results

### 3.1. Study Population and Intestinal Parasitic Infections

**Figure 1:**
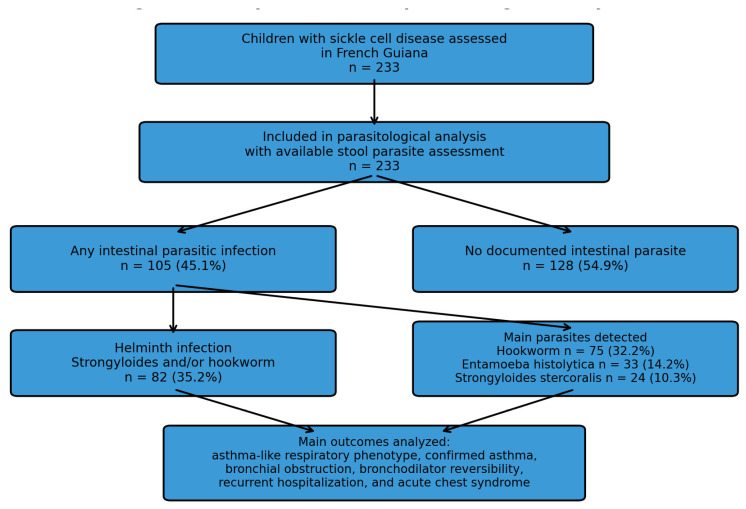
Study flowchart for parasitological analysis. Among 233 children included in the study, 105 (45.1%) had at least one documented intestinal parasitic infection, whereas 128 (54.9%) had no documented intestinal parasite. Helminth infection, defined as *Strongyloides stercoralis* and/or hookworm infection, was observed in 82 children (35.2%).

During the January 2022 to December 2025 study period, a total of 233 children and adolescents younger than 18 years with SCD were included in the analysis from a broader multicenter pediatric SCD follow-up population of approximately 588 children in French Guiana. Overall, 105 children (45.1%) had at least one documented intestinal parasitic infection, while 128 (54.9%) had no documented intestinal parasite in the available records. Helminth infection, defined as *Strongyloides stercoralis* and/or hookworm infection, was observed in 82 children (35.2%). Hookworm was the most common parasite, identified in 75 children (32.2%), followed by *Entamoeba histolytica* in 33 children (14.2%) and *Strongyloides stercoralis* in 24 children (10.3%). Children with intestinal parasitic infection were slightly older than those without a documented infection (9.2 +/− 3.8 versus 8.1 +/− 3.4 years; *p* = 0.026). Baseline characteristics according to infection status are presented in Table 1, and the study flow is summarized in Figure 1.

### 3.2. Intestinal Parasitic Infections and Respiratory Outcomes

An asthma-like respiratory phenotype was observed in 32/105 children with intestinal parasitic infection (30.5%) compared with 44/128 children without intestinal parasitic infection (34.4%). Confirmed asthma showed the same counts because, in this routine-care dataset, the children classified as having an asthma-like respiratory phenotype were also recorded as having physician-diagnosed asthma. Bronchial obstruction, bronchodilator reversibility, recurrent hospitalization, and history of ACS were not significantly different between groups. Crude comparisons of respiratory and clinical outcomes are presented in Table 2, and adjusted regression results are summarized in Table 3.

### 3.3. Species-Specific Descriptive Findings

Descriptive parasite-specific findings are reported because peer review requested species-specific information. Because many children had sparse outcome cells and possible coinfections, species-specific multivariable regression models were not considered reliable. The descriptive analyses did not reveal a clear signal suggesting that hookworm, *Strongyloides stercoralis*, or *Entamoeba histolytica* individually identified children with asthma-like respiratory phenotype, recurrent hospitalization, or ACS. Species-specific descriptive findings are presented in Table 4.

### 3.4. Exploratory Subgroup Considerations

Age and SCD genotype were accounted for in the main adjusted models. Exploratory subgroup descriptions by age group and SCD genotype did not provide sufficiently stable data for separate regression models. In particular, younger children and children with HbSS or HbSβ0 disease may have higher respiratory vulnerability, but the present sample size was insufficient to determine whether the parasite-respiratory relationship differed across these subgroups. Therefore, heterogeneous subgroup effects cannot be excluded.

## 4. Discussion

The main finding of this study is that documented intestinal parasitic infections were common among children and adolescents with SCD in French Guiana, affecting nearly half of the analytic sample, but they were not associated with asthma-like respiratory phenotypes or selected clinical morbidity outcomes in crude or adjusted analyses. These findings suggest that, in this tropical pediatric SCD population, routine parasitological status alone could not identify children with respiratory morbidity. However, because parasitological testing and respiratory assessments were performed during routine care rather than through a standardized research protocol, the absence of associations should be interpreted cautiously.

Respiratory morbidity in SCD is multifactorial. Children with SCD may develop respiratory symptoms due to airway inflammation, bronchial obstruction, impaired lung growth, hypoxemia, acute chest syndrome, infection, or vascular lung disease. Recent studies have shown that abnormal lung function is common in pediatric SCD, although obstructive, restrictive, and mixed patterns vary according to age, setting, and testing methods [11,12,13,14,15,16,17].

Environmental context remains clinically relevant in children with SCD, but our dataset only captured limited proxies for environmental exposure, including rural residence, site of follow-up, environmental tobacco smoke exposure, allergen sensitization when available, and intestinal parasite status. In the present study, parasite status should only be interpreted as one routine-care infectious/environmental marker and not as a comprehensive measure of environmental exposure. Recent studies also indicate that air pollution and socioeconomic context can influence pulmonary and acute clinical outcomes in children and adults with SCD [18,19,20,21].

The relationship between helminth infections and asthma or allergic disease is complex. Some helminths may promote Th2 responses, eosinophilia, IgE production, and airway hyperresponsiveness, whereas chronic infections may also induce regulatory pathways that dampen allergic inflammation. Recent data remain heterogeneous. Our findings are consistent with this mixed literature. In our cohort, intestinal parasitic infection was not positively associated with confirmed asthma, bronchial obstruction, or bronchodilator reversibility, suggesting that any immunomodulatory effect of parasites may be too heterogeneous, species-specific, or context-dependent to be captured by a binary “any parasite” variable. Epidemiological studies have likewise reported heterogeneous associations between helminth infection and childhood asthma or allergic disorders [22,23,24].

Accordingly, the mechanistic statements in this manuscript should be understood as biologically plausible hypotheses rather than as mechanisms demonstrated in our cohort. The study did not analyze eosinophil counts, total IgE, parasite-specific IgE, regulatory cytokines such as IL-10 or TGF-beta, or other immune biomarkers. This absence of immunological phenotyping prevents any direct assessment of Th2 polarization, regulatory immune responses, or gut-lung axis pathways in the included children. Immunological profiling studies in pediatric SCD further support the complexity of immune phenotypes in this population [25].

Another possible explanation is that the parasite-asthma relationship may depend on parasite species, parasite burden, chronicity, host age, nutritional status, microbiome composition, and background allergic sensitization. Our study grouped parasites into broad categories, including any intestinal parasitic infection and helminth infection. Such definitions are clinically pragmatic but may mask species-specific effects. For example, hookworm-related anemia, *Strongyloides stercoralis* autoinfection, and infections with protozoa such as *Entamoeba histolytica* may have different biological and clinical implications. Future studies should therefore consider parasite intensity, repeated testing, eosinophil counts, total and specific IgE, and inflammatory biomarkers to better characterize parasite-related immune profiles.

The same caution applies to subgroup interpretation. Age and SCD genotype may modify both parasite exposure and respiratory vulnerability, particularly in younger children and in those with HbSS or HbSβ0 disease. Our adjustment strategy accounted for age and genotype in the overall models, but the sample size was insufficient for reliable stratified regression models. Therefore, heterogeneous effects across age or genotype groups cannot be excluded.

The gut-lung axis provides a possible biological framework for future research, but it remains speculative in relation to the present findings. Environmental microbial exposure, intestinal microbiota, and airway microbiota have been implicated in allergic disease and asthma [26,27,28], and these mechanisms could be relevant in SCD because of baseline inflammation, hemolysis, endothelial dysfunction, and susceptibility to hypoxemia. However, our study did not include microbiome sequencing, quantitative parasite burden, eosinophil counts, IgE profiling, or direct measurement of inflammatory mediators. Therefore, the present results cannot support mechanistic conclusions about the gut-lung axis; they only indicate that routine stool parasitology status was not associated with the respiratory outcomes analyzed.

Strongyloidiasis deserves particular attention in tropical settings because *Strongyloides stercoralis* may persist for decades through autoinfection and can cause severe disease in immunosuppressed individuals [29]. Although our study did not find a clear association between intestinal parasitic infection and respiratory outcomes, screening and treatment of strongyloidiasis remain clinically important, especially before corticosteroid exposure or immunosuppression. This is particularly relevant in children with asthma-like symptoms who may receive systemic corticosteroids during exacerbations or severe respiratory events. Thus, the clinical value of parasite screening may lie less in predicting asthma-like phenotypes and more in preventing parasite-related complications in selected high-risk situations.

The representativeness of the analytic sample should be considered in relation to the broader pediatric SCD population in French Guiana. Previous multicenter local data reported that approximately 588 children were followed for SCD by the participating centers and the estimated physician-diagnosed asthma rate was 8.9% among children with major SCD genotypes [30]. This estimate may be lower than rates observed under active screening strategies because routine clinical diagnosis can miss mild or under-recognized asthma. A separate local cohort of children referred for allergology assessment also illustrates how active respiratory evaluation may identify asthma beyond routine diagnosis [31].

This study has several strengths. It focuses on an underrepresented tropical Amazonian setting and examines intestinal parasitic infections in a clinically vulnerable pediatric SCD population. It also distinguishes respiratory outcomes, including confirmed asthma, bronchial obstruction, and bronchodilator reversibility, from broader clinical morbidity outcomes such as recurrent hospitalization and ACS. However, several limitations must be acknowledged. The cross-sectional and retrospective design prevents causal inference and does not establish temporality between parasitic infection and respiratory phenotype. Because parasitological testing, spirometry, and asthma ascertainment were performed during routine care, the study may be affected by selection bias and ascertainment bias. The selected covariates did not fully capture SCD severity, baseline hemoglobin, prior ACS frequency, asthma treatment, corticosteroid exposure, eosinophilia, detailed indoor and outdoor environmental exposures, parasite burden, or repeated parasitological testing. The composite asthma-like respiratory phenotype was pragmatic and may overlap substantially with physician-diagnosed asthma in this dataset. Finally, the sample size limited the ability to detect small differences or parasite species-specific effects.

Additional limitations include the absence of standardized repeated parasitological testing, absence of parasite-burden quantification, possible false-negative stool examinations, absence of eosinophil and IgE-based immune phenotyping, lack of data on deworming history, and inability to adjust for household sanitation, crowding, nutritional status, or detailed socioeconomic deprivation. These limitations may have reduced the ability to detect parasite-specific, immune-mediated, or subgroup-specific associations.

## 5. Conclusions

Documented intestinal parasitic infections were common among children and adolescents with SCD in French Guiana but were not independently associated with asthma-like respiratory phenotypes, recurrent hospitalization, or ACS in this routine-care analytic sample. These findings do not exclude species-specific, immunological, or longitudinal effects of parasites but they suggest that routine parasitological status alone cannot identify children with respiratory morbidity in this cohort. Future prospective studies should combine standardized repeated parasitological testing, parasite burden assessment, eosinophil counts, total and specific IgE profiles, immune phenotyping, microbiome analysis, detailed environmental assessment, deworming history, and longitudinal respiratory follow-up to clarify whether specific parasites or immune profiles influence respiratory vulnerability in children with SCD.

## Figures and Tables

**Table 1:** Baseline characteristics according to intestinal parasitic infection status.

Characteristic	Total (n = 233)	Infected (n = 105)	Non-Infected (n = 128)	*p*-Value
Age, years, mean +/− SD	8.6 +/− 3.7	9.2 +/− 3.8	8.1 +/− 3.4	0.026
Male sex	108 (46.4%)	54 (51.4%)	54 (42.2%)	0.187
HbSS genotype	174 (74.7%)	82 (78.1%)	92 (71.9%)	0.293
Hydroxyurea treatment	191 (82.0%)	86 (81.9%)	105 (82.0%)	1.000
Rural residence	174 (74.7%)	72 (68.6%)	102 (79.7%)	0.069
Saint-Laurent-du-Maroni residence	136 (58.4%)	64 (61.0%)	72 (56.2%)	0.506
Environmental tobacco smoke exposure	40 (17.2%)	14 (13.3%)	26 (20.3%)	0.168
Dermatophagoides sensitization	175 (75.1%)	79 (75.2%)	96 (75.0%)	1.000
Blomia tropicalis sensitization	197 (84.5%)	87 (82.9%)	110 (85.9%)	0.586
Polysensitization	221 (94.8%)	101 (96.2%)	120 (93.8%)	0.554

Note: *p*-values were calculated using Fisher exact test for categorical variables and Welch *t*-test for age.

**Table 2:** Respiratory and clinical outcomes according to intestinal parasitic infection status.

Outcome	Infected (n = 105)	Non-Infected (n = 128)	Crude OR	95% CI	*p*-Value
Asthma-like respiratory phenotype	32 (30.5%)	44 (34.4%)	0.84	0.48–1.45	0.576
Confirmed asthma	32 (30.5%)	44 (34.4%)	0.84	0.48–1.45	0.576
Bronchial obstruction	33 (31.4%)	45 (35.2%)	0.85	0.49–1.46	0.579
Bronchodilator reversibility	33 (31.4%)	42 (32.8%)	0.94	0.54–1.63	0.888
>2 hospitalizations	37 (35.2%)	45 (35.2%)	1.00	0.58–1.72	1.000
History of acute chest syndrome	33 (31.4%)	35 (27.3%)	1.22	0.69–2.15	0.563

**Table 3:** Logistic regression models for the association between intestinal parasitic infection and respiratory or clinical outcomes.

Outcome	Exposure	Adjusted OR (95% CI)	*p*-Value	Adjustment Variables
Asthma-like respiratory phenotype	Any intestinal parasitic infection	0.94 (0.51–1.72)	0.844	Age, sex, genotype, hydroxyurea, rural residence, site, environmental tobacco smoke
Recurrent hospitalization	Any intestinal parasitic infection	No independent association	—	Same variables
History of ACS	Any intestinal parasitic infection	No independent association	—	Same variables
Confirmed asthma	Any intestinal parasitic infection	No positive association	—	Same variables
Bronchial obstruction	Any intestinal parasitic infection	No positive association	—	Same variables
Bronchodilator reversibility	Any intestinal parasitic infection	No positive association	—	Same variables

**Table 4:** Species-specific descriptive findings.

Parasite Species	n/N (%)	Asthma-Like Respiratory Phenotype	Recurrent Hospitalization	History of ACS	Comment
*Hookworm*	75/233 (32.2%)	Descriptive comparison only	Descriptive comparison only	Descriptive comparison only	Effect estimates unstable if adjusted
*Strongyloides stercoralis*	24/233 (10.3%)	Descriptive comparison only	Descriptive comparison only	Descriptive comparison only	Small subgroup; clinically important before corticosteroids
*Entamoeba histolytica*	33/233 (14.2%)	Descriptive comparison only	Descriptive comparison only	Descriptive comparison only	May reflect fecal-oral/sanitation exposure
*Any helminth*	82/233 (35.2%)	No clear positive signal	No clear positive signal	No clear positive signal	Analyzed as secondary exposure

## Data Availability

The dataset analyzed during the current study is not publicly available because it contains sensitive clinical information from children with SCD. De-identified data may be made available from the corresponding author upon reasonable request and subject to institutional and regulatory approval.

## Abbreviations

The following abbreviations are used in this manuscript:

ACS	acute chest syndrome
aOR	adjusted odds ratio
CI	confidence interval
FEV1	forced expiratory volume in one second
FEV1/FVC	ratio of forced expiratory volume in one second to forced vital capacity
OR	odds ratio
SCD	sickle cell disease
SD	standard deviation
STH	soil-transmitted helminths
STROBE	Strengthening the Reporting of Observational Studies in Epidemiology
